# Traumatic intercostal arterial bleeding controlled with a novel surgical technique: a case report

**DOI:** 10.1186/1752-1947-6-318

**Published:** 2012-09-25

**Authors:** Simo Miettinen, Tapio Hakala

**Affiliations:** 1 Department of Surgery, North Karelian Central Hospital, Tikkamaentie 16, Joensuu, 80210, Finland

**Keywords:** Bleeding, Hemothorax, Surgery, Emergency, Thoracotomy, Trauma, Blunt

## Abstract

**Introduction:**

A blunt thoracic trauma may cause arterial bleeding requiring operative treatment or endovascular embolization or endovascular aortic stenting. A novel damage control technique to stop such bleeding is presented.

**Case presentation:**

We present the case of an 82-year-old Caucasian man who experienced rib fractures I-VII on the left side and bleeding from damaged intercostal arteries after a blunt thoracic trauma. Emergency thoracotomy was performed.

**Conclusions:**

Effective hemostasis was achieved by using a rolled surgical swab and inserting it against the chest wall next to the aorta with sutures pulled through the intercostal muscles and then sutured to the back side of the patient. The patient died four days after the surgery due to a head injury sustained in the car crash.

## Introduction

Intrathoracic hemorrhage is a common finding associated with blunt thoracic trauma, especially in older people [1]. It is often related to traumatic intercostal artery (ICA) injuries associated with rib fractures [2,3]. When the bleeding is self-limiting, chest tube insertion and fluid resuscitation represent the treatments of choice. If the conservative treatment is not sufficient and the bleeding continues, then exploratory thoracotomy or endovascular embolization or endovascular aortic stenting may be treatment options [4-6]. Bleeding from the posterior intercostal artery deep in the chest can be very difficult to control [7]. We describe a novel damage control technique to stop the bleeding from injured intercostal arteries.

## Case presentation

**Figure 1:**
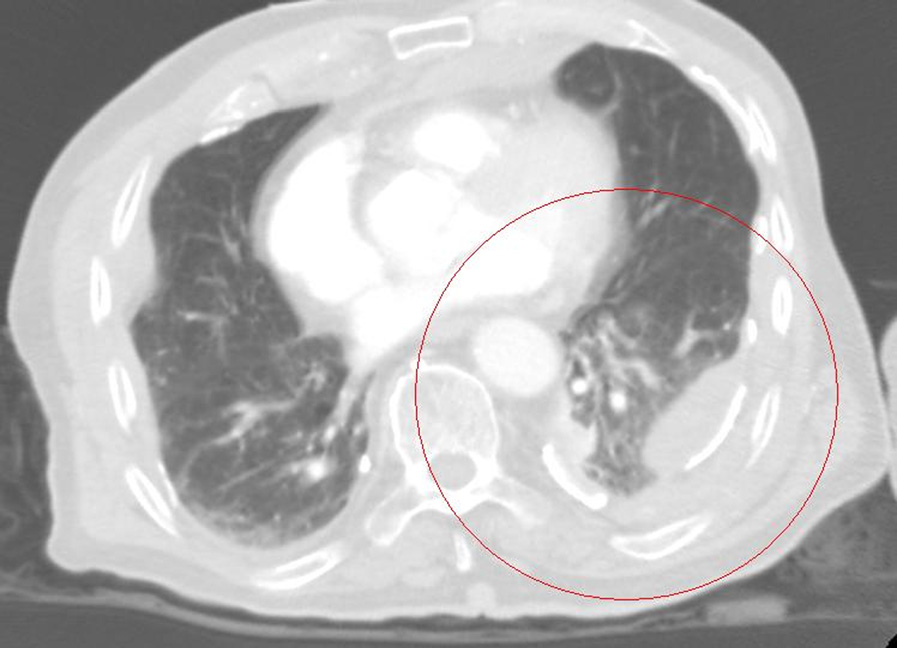
The computerized tomography shows left sided hemothorax (red circle) and extravasation of contrast medium to the thoracic cavity.

We present the case of an 82-year-old Caucasian man with a history of coronary artery disease. He was the driver of a vehicle involved in an accident in which two cars collided while travelling at 80 to 100km/hour. He was conscious immediately after the car crash and his Glasgow Coma Scale was 15. On arrival at the Accident and Emergency Department (EA) he complained of neck and chest pain but was hemodynamically stable. Trauma computerized tomography (CT) revealed an unstable fracture of the second cervical vertebrae, dislocated rib fractures III-VII on the right side, dislocated rib fractures I-VII on the left side and a fractured sternum. There was also left-sided hemothorax with extravasation of the contrast medium (Figure 1). Trauma CT did not reveal any abdominal organ injuries. Soon after the trauma CT had been performed, the patient suddenly became pulseless. An endotracheal tube was inserted and left sided thoracotomy was performed in the EA. A large pool of blood was encountered in the left thoracic cavity. The blood was evacuated and open cardiac massage initiated. A pulse was restored and the patient was transferred to the operating theater (OR).

**Figure 2:**
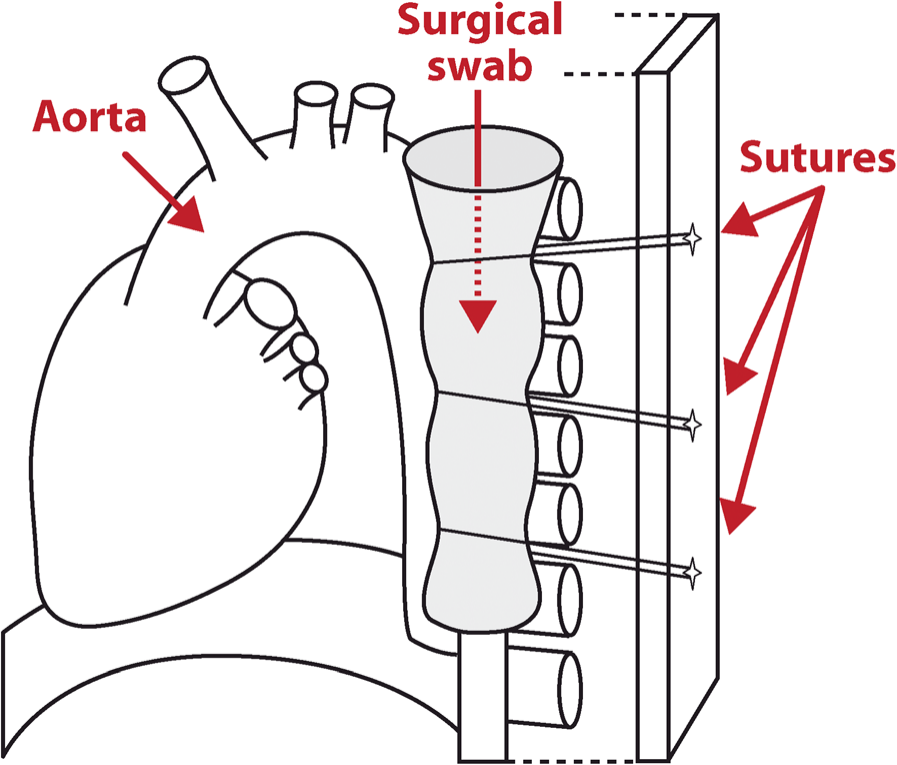
**A schematic figure.** A surgical swab inserted adjacent to the aorta and secured with three sutures.

During surgery, it became obvious that there was continuous arterial bleeding from several ICAs at the level of ribs V-VII on the left side of the thoracic cavity. The bleeding source was determined to be close to the aortic origin of these arteries. First, several hemostatic options were attempted but there was no success using sutures, hemostatic clips, electrocautery and tissue glue and bleeding continued unabated despite several attempts with these techniques. Finally, hemostasis was achieved by using a surgical swab which was rolled tight and inserted over the assumed bleeding site next to the descending aorta. Three sutures were pulled through the posterior thoracic wall from inside to outside. Sutures were then applied around the surgical swab and pulled to the back of the patient and then tied. These sutures compressed the surgical swab around the bleeding sites and the compression succeeded in stopping the bleeding (Figure 2). Thoracotomy was closed and a halo vest was set to stabilize the fracture of the second cervical vertebrae.

He was transferred to the intensive care unit (ICU) but soon his hemoglobin value dropped and his abdomen became swollen. He was taken back to the OR and laparotomy was performed. Rupture and bleeding from the mesentery of the transverse colon were detected. Transverse colectomy and transverse colostomy were undertaken. The patient was treated in the ICU during the following days. There was no further post-operative bleeding from the thoracic or abdominal cavities and he was hemodynamically stable. However, he remained unconscious. Repeated head CT revealed the presence of traumatic intracerebral hemorrhage. He died on the fourth post-operative day due to this head injury.

## Discussion

Exploratory thoracotomy has traditionally been the treatment of choice for persistent hemothorax after blunt thoracic trauma. In recent years, the feasibility and reliability of transcatheter arterial embolization have been demonstrated to be beneficial in controlling the bleeding from an injured ICA [6,8]. Endovascular aortic stenting has also been used successfully to control bleeding from an ICA [4]. In our case, these types of endovascular treatments were not viable options because the bleeding was so intense that an emergency thoracotomy had to be performed.

Bleeding from an ICA deep in the posterior chest wall may be difficult to control surgically [7]. There was bleeding from several ICAs very close to the thoracic aorta of our patient. There are no straightforward surgical methods to control this kind of arterial bleeding. In this patient, it was impossible to stop the bleeding with traditional surgical techniques. In order to prevent exsanguination, damage control hemostasis was achieved with the surgical technique we have described above. In our case, this novel technique worked well in controlling the bleeding from the intercostal arteries. We had planned to perform a definitive repair with an aortic stent-graft and to remove the surgical swab during re-thoracotomy some days later. Our patient died due to a head injury incurred in the car crash on the fourth post-operative day.

## Conclusions

The damage control technique we describe is an option when the bleeding from ICAs is difficult to control by traditional surgical procedures.

## Consent

Written informed consent for publication could not be obtained from the patient’s next-of-kin despite all reasonable attempts. The case is important for public health and every effort has been made to conceal the identity of our patient. There is no reason to believe that our patient’s next-of-kin would object to this publication.

## Competing interests

The authors declare that they have no competing interests.

## Authors’ contributions

TH performed surgery and SM assisted in the surgery and both were major contributors in writing the manuscript. All authors read and approved the final manuscript.
